# Quantitative Determination of 15 Active Components in *Lepidium meyenii* with UHPLC-PDA and GC-MS

**DOI:** 10.1155/2021/6333989

**Published:** 2021-09-01

**Authors:** Yao-qing Xu, San-yang Qiao, Zi-qian Wang, Meng-yao Cui, Dao-peng Tan, Hong Feng, Xing-sha Mei, Geng Li, Long Cheng

**Affiliations:** ^1^School of Pharmacy, Zunyi Medical University, Zunyi, Guizhou 563006, China; ^2^Beijing LabTech Instrument Co.,Ltd., Beijing 101312, China; ^3^Jiangxi Drug Inspection Centre, Nanchang, Jiangxi 330029, China; ^4^Jiangxi University of Traditional Chinese Medicine, Nanchang, Jiangxi 330004, China; ^5^State Key Laboratory of Proteomics, Beijing Proteome Research Center, Beijing Institute of Radiation Medicine, Beijing 102206, China; ^6^Institute of Medicinal Plant Development, Chinese Academy of Medical Sciences & Peking Union Medical College, Beijing 100193, China

## Abstract

In this study, a method using ultrahigh-performance liquid chromatography with photodiode array (UHPLC-PDA) was established and validated for the simultaneous quantification of 10 active components, including eight macamides and two glucosinolates, in *Lepidium meyenii* (maca). A gas chromatographic mass spectroscopy (GC-MS) method was used to determine the levels of three benzyl isothiocyanates and two sterols in maca. Liquid chromatographic separation was achieved on a Waters Acquity UHPLC HSS T3 column (2.1 mm × 100 mm, 1.8 *μ*m) with gradient elution over 15 min. The mobile phase was (B) acetonitrile-(A) 10 mM aqueous ammonium phosphate, and the detection wavelength was 210 nm. The gas chromatographic separation was performed on an SH-Rxi-1 MS column, and the ionization mode was electron ionization (EI). Two methods were confirmed to have desirable precision (RSD < 1.58%), repeatability (RSD < 1.97%), stability (RSD < 1.76%), and good linearity (*R*^2^ ≥ 0.999) within the test range. The recoveries were in the range of 96.79–109.99%, with an RSD below 2.39%. We applied the established methods and successfully analyzed 15 compounds in maca processed under different drying conditions, providing a comprehensive reference for maca processing method of development. In summary, this study provided two rapid and effective methods for the quantification of 15 active components, which contributed to the in-depth maca quality control and provided a reference for the development of maca products.

## 1. Introduction

*Lepidium meyenii* (maca), a well-known nutritious and healthy vegetable, was applied widely in the folk medicine in the South American Andes region, as an enhanced energy agent, improving fertility and sexual function [1]. It was approved as a new food ingredient by the Ministry of Health of China in 2011. Maca, the Peruvian herb, contains mainly alkaloids, mustard glycosides, volatile oils, polyphenols, and macaenes [2]. Various bioactivities of maca include enhanced reproductive health, antifatigue, antioxidation, neuroprotection, antimicrobial activity, anticancer, hepatoprotection, immunomodulation, and improving skin health and the function of digestive system [3–6]. Plant genetics, botanical parts, processing, extraction, and experimental protocols represent the major factors affecting the chemical composition, physicochemical attributes, and health effects of maca-based products. However, the quality control related to its efficacy appears to be lacking in the current literature.

A variety of secondary metabolites, such as alkaloids, glucosinolates and sterols, are thought to be closely related to these unique effects of maca. Among them, a variety of compounds in maca have been identified, including macamides (linoleamide, linamide, and benzyl-hexadecyl amide), glucosinolates (benzyl glucosinolate and M-methoxybenzyl glucosinolate), and volatile oils (benzyl isothiocyanate and phenylacetonitrile) [7–9]. Macamides are the main active components of maca that inhibit acetylcholinesterase, antifatigue and free radical scavenging, and *N*-benzyl-hexadecanamide is the active maca ingredient that is responsible for the antifatigue effects [10–12]. Furthermore, glucosinolates and benzyl isothiocyanate show high anticancer activity [13].

After being introduced to China as a new health food ingredient, maca has been widely cultivated in Yunnan, Tibet, Xinjiang, and Sichuan [14]. Recently, maca has been rapidly developed in the field of food, health products, and cosmetics. The preparations of maca are produced mainly as maca wine, maca wolfberry capsules, maca oral liquid, maca astragalus and American ginseng tablets, etc. [15, 16]. Product innovation and diversification in food and non-food utilization of different parts of maca to maximize the value perceptions are important and urgent.

In order to ensure the quality and good reputation of maca food and maca product, a systematic raw material quality control is particularly necessary and urgent. The current quality evaluation methods for maca essentially rely on determining the content of a single compound (macamide or glucosinolate), the contents of a few compounds or the total content of a class of compounds as the quality control indicator [17, 18]. These methods cannot accurately assess the overall quality of maca or clearly distinguish its characteristics. Hence, a more rigorous quality evaluation method is urgently required.

To date, a variety of analytical and quantitative methods have been employed for the analysis of maca. UHPLC coupled with PDA, a novel rapid, sensitive and high-resolution technique which has been used to quantify chemical components in several different complex matrices, has been widely applied to analyze and quantify multitarget components in medicinal herbs [19]. GC-MS can detect, identify, and quantify trace chemical substances in complex matrices, and it is considered the “gold standard” for the analysis of volatile organic compounds (VOCs) and semivolatile organic compounds (SVOCs) [20]. The present study utilizes chemical holography research strategy [21] and established two rapid, accurate, and precise UHPLC-PDA and GC-MS methods for the estimation of glucosinolates, benzyl isothiocyanates, macamides, and sterols in maca samples, providing a theoretical research basis for maca quality control. Moreover, applying the established quantitative methods, we analyzed the influences of different drying methods (oven drying at 40°C, 60°C, and 80°C and natural drying 12–25°C) on the contents of 15 components during postharvest of Lijiang maca to provide a reference for optimizing maca processing methods.

## 2. Materials and Methods

### 2.1. Equipment and Materials

An electronic balance (BSM220.4) was purchased from Shanghai Zhuojing Electronic Technology Co., Ltd. (Shanghai, China), and an ultrasound machine was obtained from Kunshan Ultrasonic Instruments Co., Ltd. The microtome and drying oven were purchased from Yongkang Bo'ou Hardware Products Co., Ltd. (Yongkang, China) and the Wujiang Yonglian Mechanical Equipment Factory (Wujiang, China), respectively. The first batch (6 kg) of fresh maca was collected from Yulong Mountain, Lijiang, Yunnan, China, in mid-February 2019, and the second batch (3 kg) was collected in mid-March 2019. The maca samples were identified by Professor Xue-Yong Wang (Beijing University of Chinese Medicine) as the rhizome of cruciferous maca (*Lepidium meyenii*) plants, and they were sealed with soil after collection and cryopreserved at −27°C.

### 2.2. Chemical Standards and Reagents

**Figure 1:**
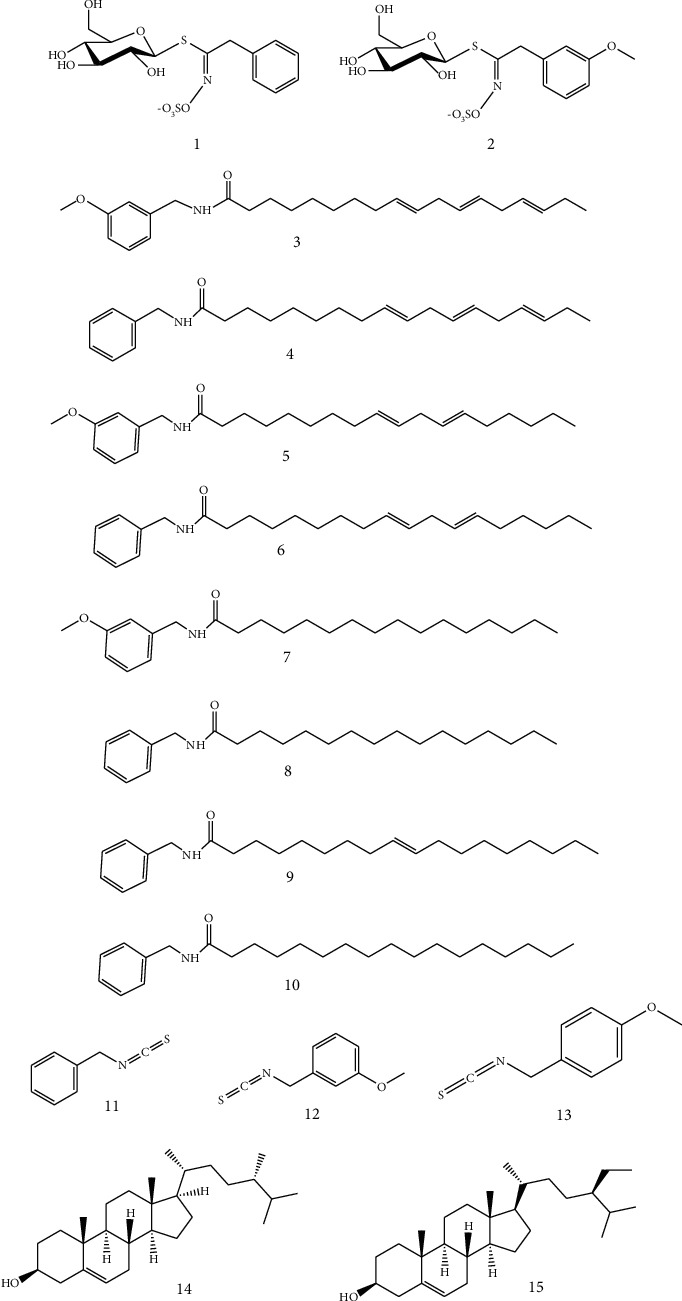
The chemical structures of 15 refence substances. (1) Benzyl glucosinolate (BGLU), (2) *M*-methoxybenzyl glucosinolate (MGLU), (3) *N*-(m-methoxybenzyl)-octadecatrienamide (MOCT), (4) *N*-benzyl-9Z,12Z,15Z-octadecatrienamide (BOCT), (5) *N*-(m-methoxybenzyl)-octadecadienamide (MOCD), (6) *N*-benzyl-9Z,12Z-octadecadienamide (BOCD), (7) *N*-(3-m-methoxybenzyl)-hexadecanamide (MHEX), (8) *N*-benzyl-hexadecanamide (BHEX), (9) *N*-benzyl-9Z-octadecenamide (BOCN), (10) *N*-benzyl-heptadecanamide (BHEP), (11) benzyl isothiocyanate (BISO), (12) 3-methoxybenzyl isothiocyanate (MISO), (13) 4-methoxybenzyl thiocyanate (MTHI), (14) campesterol (CAM), and (15) sitosterol (SIT).

The *N*-(m-methoxybenzyl)-octadecadienamide, *N*-(m-methoxybenzyl)-octadecatrienamide, *N*-benzyl-9Z,12Z,15Z-octadecatrienamide, *N*-(3-m-methoxybenzyl)-hexadecanamide, *N*-benzyl-9Z,12Z-octadecadienamide, *N*-benzyl-hexadecanamide, *N*-(m-methoxybenzyl)-9Z-octadecenamide, *N*-benzyl-heptadecanamide, benzyl glucosinolate, *M*-methoxybenzyl glucosinolate, benzyl isothiocyanate, 4-methoxybenzyl isothiocyanate, and 3-methoxybenzyl thiocyanate reference standards were purchased from Wuhan Huashite Industrial Biotechnology Development Co., Ltd. (Wuhan, China). Campesterol and sitosterol were purchased from Nanchang Beta Biotechnology Co., Ltd. (Nanchang, China). The purity of the above compounds was greater than 98%, meeting requirements for HPLC. The chemical structures of the 15 reference compounds are shown in Figure 1. HPLC-grade ethyl acetate and analytical-grade ammonium phosphate were purchased from Shanghai Macklin Biochemical Co., Ltd. (Shanghai, China), analytical-grade methanol was obtained from Tianjin Damao Chemical Reagent Factory (Tianjin, China), and HPLC-grade acetonitrile was obtained from Thermo Fisher Scientific China Co., Ltd. (New York, USA). Wahaha purified water was commercially available.

### 2.3. Quantitative Analysis of Macamides and Glucosinolates with UHPLC-PDA

#### 2.3.1. Liquid Chromatographic Conditions

Analyses were performed by a Waters UHPLC Accuracy system (Waters Crop, Milford, MA, USA) equipped with a binary solvent system, automatic sample injection, constant-temperature sample manager, photodiode array detector, and Empower 3 chromatography workstation. All separations were performed on a Waters Acquity UHPLC HSST3 column (2.1 × 100 mm, 1.8 *μ*m), and the detection wavelength was 210 nm. The mobile phases consisted of (B) acetonitrile and (A) 10 mM aqueous ammonium phosphate using the following gradient elution: 2%–16% B at 0–6 min, 16%–80% B at 6–8.5 min, 80%–95% B at 8.5–11.5 min, 95% B at 11.5–14 min, and 95%–2% B at 14–15 min. The solvent flow rate was set at 0.3 mL/min. The injection volume was 2 *μ*L. In addition, the column temperature and sample temperature were maintained at 35°C and 4°C, respectively.

#### 2.3.2. Preparation of Reference Solutions

*N*-Benzyl-(9z)-octadecenamide, *N*-(3-m-methoxy-benzyl)-hexadecanamide, *N*-benzyl-hexadecanamide, and *N*-benzyl-heptadecanamide were accurately weighed 5.86 mg, 7.99 mg, 5.21 mg, and 5.82 mg dissolved in methanol and diluted to 50 mL in a volumetric flask to prepare reference stock solutions with concentrations of 0.117 mg/mL, 0.160 mg/mL, 0.104 mg/mL, and 0.116 mg/mL, respectively. To prepare reference stock solutions with concentrations of 0.179 mg/mL, 0.195 mg/mL, 0.202 mg/mL, 0.152 mg/mL, 0.251 mg/mL, and 0.320 mg/mL, 4.47 mg of *N*-(m-methoxybenzyl)-octadecadienamide, 4.88 mg of N-(m-methoxybenzyl)-octadecatrienamide, 5.05 mg of *N*-benzyl-9Z,12Z,15Z-octadecatrienamide, 3.79 mg of *N*-benzyl-9Z,12Z-octadecadienamide, 6.27 mg of benzyl glucosinolate, and 8.01 mg of *M*-methoxybenzyl glucosinolate were accurately weighed and dissolved in methanol, and the solutions were brought to 25 mL in a volumetric flask.

#### 2.3.3. Pretreatment of Fresh Maca Samples

First, fresh maca samples with no visible damage, decay, or lesions were rinsed with clean water, and the main root and fibrous root were sorted. Next, the washed fresh maca samples were cut into 1-mm-thick maca slices by microtome, weighed, and recorded. Finally, each maca slice was weighed and then dried at room temperature (12–25°C), sampling after 14 d. After getting dried, the maca moisture content was less than 5%.

#### 2.3.4. Preparation of Sample Solutions

The naturally dried maca samples were crushed into powder by a pulverizer. One gram of accurately weighed powder was mixed with 10 mL of 75% aqueous methanol, in an Erlenmeyer flask, and its weight was recorded. Ultrasonic extraction was performed for 30 min, in a 15 mL Erlenmeyer flask. After that, the sample was cooled at room temperature, and the weight was recorded. Then, 75% aqueous methanol was added to make up the difference in weight. After incubating for 30 sec, the supernatant was filtered through a 0.2 *μ*m filter membrane, and the concentration in the solution was measured.

### 2.4. Quantitative Analysis of Benzyl Isothiocyanates and Sterols with GC-MS

#### 2.4.1. GC Conditions

Analyses were performed by a gas chromatography mass spectrometry system (GCMS-QP2020; single quadrupole, Shimadzu) with the NIST17 mass spectrometry search database. The analytes were separated by injecting 1 *μ*L of a sample onto an SH-Rxi-1 MS column (30 m × 0.25 mm × 0.25 *μ*m). The carrier gas was helium (purity >99.99%), and the total flow rate was set to 9 mL/min. The column and purge flow rates were set to 1 mL/min and 3 mL/min, respectively. The split ratio was 5 : 1, and the linear speed was 36.5 cm/sec. The program of column temperature was set from 60°C up to 200°C at a rate of 10°C/min and continue up to 280°C with a rate of 30°C/min (keeping for 10 min).

#### 2.4.2. MS Conditions

The ionization mode was electron ionization (EI), and the ion source temperature was set at 250°C. Scan was used as the acquisition mode, and the interval was 0.3 sec with a scanning speed of 1666. In addition, the solvent delay was set to 2 min, and NIST 18 was used as the search library.

#### 2.4.3. Preparation of Reference Standard Solutions

To prepare reference stock solutions with concentrations of 0.457 mg/mL, 0.551 mg/mL, 0.606 mg/mL, 0.501 mg/mL, and 0.522 mg/mL, 4.57 mg of benzyl isothiocyanate, 5.51 mg of 4-methoxybenzyl thiocyanate, 6.06 mg of 3-methoxybenzyl isothiocyanate, 5.01 mg of campesterol, and 5.22 mg of sitosterol were accurately weighed into volumetric flasks, and 10 mL of ethyl acetate was added.

#### 2.4.4. Sample Preparation

Approximately 1 g maca powder, which had been dried naturally, was weighed and placed into a 10 mL beaker. Ten milliliters of ethyl acetate was added and the weight was recorded. The mixture was ultrasonically extracted for 60 min, and ethyl acetate was added to make up the difference in weight. The supernatant was removed through a 0.2 *μ*m filter membrane, and the concentration of volatile components in the solution was measured.

100 g maca was placed in a 500 mL round-bottom flask, and 10 volumes of pure water were added. Volatile oil was extracted and collected with a volatile oil extraction device as described in the 2020 edition of the Chinese Pharmacopoeia (General Principle 2203). The collected volatile oil was blended with 10 mL of ethyl acetate.

### 2.5. Method Performance

#### 2.5.1. Linearity, Limit of Detection (LOD), and Limit of Quantification (LOQ)

To establish calibration curves, standard stock solutions at a minimum of six concentrations were prepared by serially diluting the mixed standard solution with appropriate volumes of solvent, and all operations were repeated in triplicate. Calibration curves for the 15 bioactive compounds were generated by plotting the average peak area (*y*-axis) versus the corresponding concentration (*x*-axis) for each bioactive compound in the corresponding linear range. The limit of detection (LOD) and limit of quantification (LOQ) were determined on the basis of signal-to-noise (S/N) ratios of 3 and 10, respectively.

#### 2.5.2. Precision, Repeatability, Stability, and Recovery

Two methods were validated through precision, repeatability, stability, and accuracy studies. Precision was evaluated by six interday and intraday relative standard deviations (RSDs). The repeatability of the method was performed through the extraction and analysis of six replicates of a maca sample obtained. The stability of the samples was checked at predetermined times of 0, 4, 8, 12, 16, 20, 24, and 48 h, and the RSDs were determined for the detected concentrations of each analyte. Method accuracy was tested through spiking experiments. The recovery was calculated using the following equation: recovery = (total detected amount − original amount)/added amount × 100%.

## 3. Results and Discussion

### 3.1. UHPLC-PDA Results and Analysis

#### 3.1.1. Evaluation of Extraction Conditions

Maca contains two major types of components: liposoluble and water-soluble compounds. To develop an efficient, suitable, and convenient extraction procedure, process parameters, such as the extraction method, extraction time, extraction solvent, and material ratio, were investigated in the article.

Currently, there are many methods for the extraction of maca compounds, such as ultrasonic extraction, solvent extraction, and reflux extraction [22, 23]. Compared with other extraction methods, ultrasonic extraction is more convenient with shorter extraction time and efficiently extract rate [24]. Therefore, an ultrasonic method was selected as the final extraction method. Next, the extracts of maca were obtained with different solvents, including methanol (50% and 75% methanol in water), acetonitrile (50% and 75% acetonitrile in water), and cyclohexane. When 75% aqueous methanol or 75% aqueous acetonitrile was selected as extract solution, the concentration of macamides and glucosinolates in maca samples was higher than others. However, the sample extracted with acetonitrile produced a chromatogram with obvious interference peaks. The extraction with 75% aqueous methanol was best opinion. Next, different weight/volume ratios (w/v) were investigated: 1/10 (w/v), 1/25 (w/v), and 1/50 (w/v). The results confirmed that the ratio of 1/10 (w/v) yielded the optimal extraction efficiency. Subsequently, different extraction times (15, 30, 45, and 60 min) were compared, and the results suggested that the target bioactive components could be adequately extracted within 30 min.

#### 3.1.2. Optimization of the UHPLC Conditions

**Figure 2:**
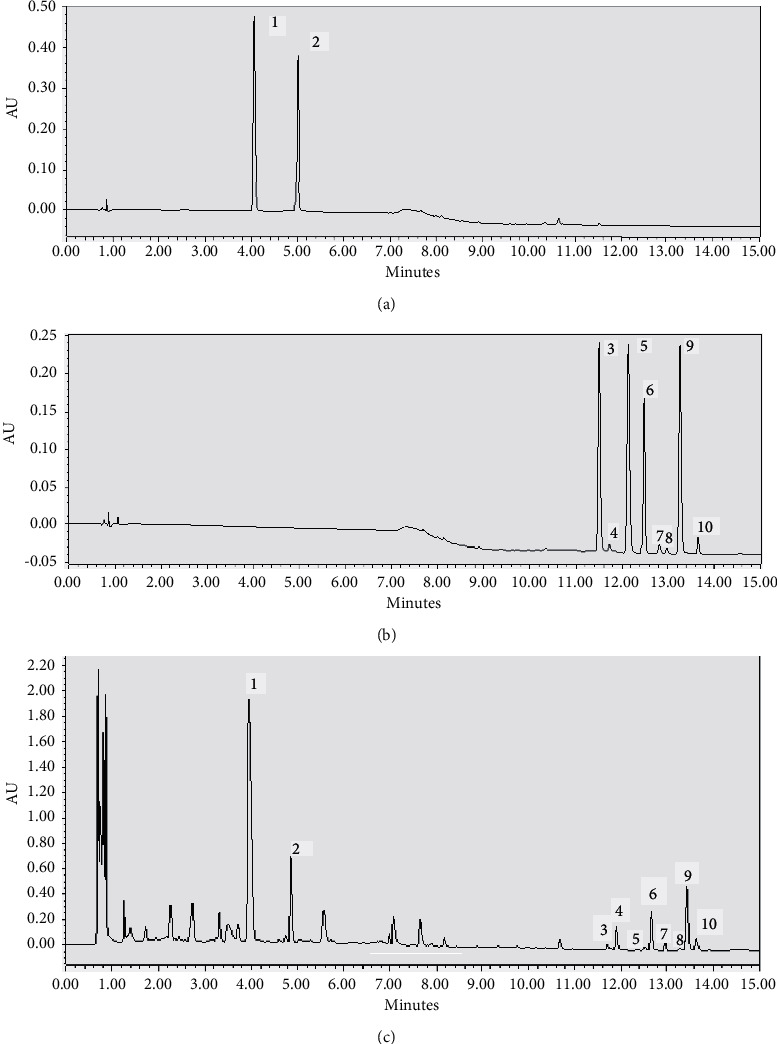
UHPLC chromatogram of (a) two glucosinolates mixed standards, (b) eight macamides mixed standards, and (c) maca test solution with (1) benzyl glucosinolate, (2) *M*-methoxybenzyl glucosinolate, (3) *N*-(m-methoxybenzyl)-octadecatrienamide, (4) *N*-benzyl-9Z,12Z,15Z-octadecatrienamide, (5) *N*-(m-methoxybenzyl)-octadecadienamide, (6) *N*-benzyl-9Z,12Z-octadecadienamide, (7) *N*-(3-m-methoxybenzyl)-hexadecanamide, (8) *N*-benzyl -hexadecanamide, (9) *N*-benzyl-9Z-octadecenamide, and (10) *N*-benzyl-heptadecanamide.

This study aimed to develop a chromatographic method with simultaneously determining 10 bioactive compounds in maca samples. Methanol and acetonitrile were compared as the organic chromatographic mobile phase. Acetonitrile not only had weak UV absorption at the low wavelengths but also had a weaker effect on baseline fluctuations than methanol. Moreover, acetonitrile could elute both liposoluble and water-soluble compounds. The proportion and composition of mobile phase were investigated, including 0.1% aqueous formic acid, 0.1% aqueous phosphoric acid, 0.1% aqueous acetic acid, and 10 mM aqueous ammonium phosphate. Acetonitrile-10 mM aqueous ammonium phosphate is preferred formula, providing a good resolution. When an Acquity HSS T3 column was used to analyze the maca samples, the chromatographic peaks of each component were well separated, and the peak shapes were sharp. Furthermore, the detection wavelength (scanned from 200 nm to 400 nm) and mobile phase flow rate (0.6, 0.5, 0.4, 0.3, and 0.2 mL/min) were examined, separately. When the detection wavelength was set to 210 nm and the flow rate was set to 0.3 mL/min, the interference from impurity peaks was minimized, as shown in Figure 2.

#### 3.1.3. Methodological Investigation

*(1) Linearity*, *LOD, and LOQ*.  Table 1 shows the regression equations, *R*^2^ values, and linear ranges for the 10 analytes. Great linear correlations were obtained between the peak areas and concentrations for each analyte. The calibration curves appeared to have good linearity (*R*^2^ ≥ 0.999) in the tested concentration ranges. The LODs and LOQs for the 10 bioactive compounds were 0.03–0.11 *μ*g/mL and 0.10–1.48 *μ*g/mL (Table 1), respectively, which suggested that the calibration curves were within adequate ranges. Thus, the method developed exhibited good sensitivity for the separation and analysis of the 10 bioactive compounds.

*(2) Precision*, *Repeatability*, *Stability, and Recoveries*.  Table 2 shows that the variations (RSD) for the intraday and interday analyses were 1.12–1.50% and 1.22–1.58%, with a repeatability of 1.50–1.97% and stability of 0.99–1.76%. These results demonstrated that the precision, repeatability, and stability of the proposed method were sufficient for the determination of the 10 compounds in maca samples. The average recovery ranged from 98.43 to 104.39%, and the RSD values were 1.20–2.23% (Table 2). Thus, the developed method exhibited good accuracy, repeatability, and stability for the simultaneous analysis of the 10 compounds. The contents (*n* = 3) of the 10 bioactive compounds in maca samples were calculated with an external standard method based on their respective calibration curves, as listed in Table 3.

### 3.2. GC-MS Results and Analysis

#### 3.2.1. Linearity, LOD, and LOQ

The established method was used to obtain complete calibration curves for 5 compounds over a relatively wide concentration range and determine the correlation coefficient. All the correlation coefficients were greater than 0.9990 (Table 4). The LODs ranged from 0.15 to 0.2 *μ*g/mL, and the LOQs ranged from 0.46 to 0.61 *μ*g/mL (Table 4). Therefore, the developed method exhibited high sensitivity for the separation and analysis of the 5 bioactive compounds.

#### 3.2.2. Precision, Repeatability, Stability, and Recovery

The variations (RSD) for the intraday and interday analyses were 1.14–1.24% and 1.09–1.21% (Table 5), respectively. The RSD for the repeatability analysis ranged from 1.57% to 1.97% (Table 5). The mixed standard solution was stable from 0 to 48 h, and the RSD value was less than 1.29% (Table 5). The average recoveries ranged from 99.43% to 109.99%, and the variations (RSD) were 1.63–2.39% (Table 5). The results above demonstrated that the precision, repeatability, stability, and recovery of the proposed method were accurate, reliable, and stable for the determination of these 5 compounds in maca samples. The contents (*n* = 3) of the 5 bioactive compounds in the maca samples were calculated with an external standard method based on their respective calibration curves, as listed in Table 6.

## 4. Applications

### 4.1. Determination of 15 Compounds after Different Drying Methods

Firstly, a random sampling of fresh maca slices was taken. The samples were divided into groups and subjected to natural drying (12–25°C) or drying in oven at 40°C, 60°C, or 80°C, and three copies were prepared for each group. The sampling time for different drying methods (oven drying at 40°C, 60°C, and 80°C and natural drying at 12–25°C) were 48 h, 24 h, 8 h, and 14 d, respectively. After getting dried, the maca moisture content was less than 5%. Then, the dried samples were extracted following the procedures described in Sections 2.3.4 and 2.4.4, and the contents of 15 compounds in maca samples dried via different methods were determined according to the two established methods.

### 4.2. Analysis of the Influences of Different Drying Methods on the Contents of Compounds

The contents of 15 compounds in maca dried via different methods are shown in Tables 7 and 8. The contents of macamides were highest in the naturally dried samples, followed by the samples dried in an oven at 40°C, but the difference between the two methods was not significant (±2%). The contents of macamides in the samples dried in an oven at 80°C were lowest. For glucosinolates and sterols, the contents showed the following trend: 80°C > 60°C > 40°C > naturally drying. There was no significant difference (±0.02%) in the content of benzyl isothiocyanate at each drying temperature.

The contents of macamides in fresh maca are low, so traditional processing after harvesting is an important way to enrich the macamide content. Compared with natural drying, drying in an oven at 40°C is much faster and less affected by the external environment. Therefore, drying in an oven at 40°C can be used as a drying method for enrichment of macamides in maca. Glucosinolates mainly exist in plant vacuoles, and their properties are stable. However, when plant tissue is damaged (such as during chewing and backlog), myrosinase is released and hydrolyzes glucosinolates. High temperature decomposes glucosinolates into different chemicals, but also inactivates myrosinase in plants. These results indicated that drying in an oven at 80°C could be the optimum drying method for glucosinolates in maca. Researchers speculate that maca can regulate endocrine functions, improve sexual functions, and increase reproductive abilities without affecting the level of reproductive hormones, which may be due to the phytoestrogen analogs in maca-phytosterols [25, 26]. The results of this study revealed that different drying temperatures have great impact on the contents of sterols. Therefore, for the enrichment of sterols, the choice of maca drying method is important. Isothiocyanates are widely found in cruciferous plants such as maca, cabbage, mustard greens, and radishes [27]. At present, there is little research on the effects of different drying methods on isothiocyanates in maca. Therefore, this research provides a reference for optimizing maca processing method to retain isothiocyanates.

## 5. Conclusion

In this study, two highly sensitive and efficient methods for the detection of 15 active components in maca were established and applied. The method showed good linearity, precision, stability, and recovery for all 15 analytes. In addition, the influence of different drying methods on the contents of the 15 compounds in maca was analyzed. The results showed that the contents of three classes of compounds varied with the drying temperatures. The drying temperature can be optimized according to the characteristics of the chemical components. Consequently, this method can be used to evaluate the quality of medicinal maca and serve as a theoretical reference for the development of medicinal maca products.

## Figures and Tables

**Table 1:** Linear equations for the 10 compounds.

Ingredient	Linear range (*μ*g/mL)	Regression equation	*R* ^2^	LOD (*μ*g/mL)	LOQ (*μ*g/mL)
BGLU	0.25∼251.80	*y* = 6133431.79*x* − 31324.33	0.9999	0.08	0.25
MGLU	0.32∼320.60	*y* = 6476016.58*x* − 16966.57	0.9999	0.11	0.32
MOCT	0.20∼195.46	*y* = 268055.79*x* + 13906.12	0.9999	0.07	0.20
BOCT	0.20∼202.40	*y* = 4667256.01*x* − 3310.29	0.9996	0.07	0.20
MOCD	0.18∼179.60	*y* = 1039482.03*x* + 21861.34	0.9992	0.06	0.18
BOCD	1.48∼152.60	*y* = 910515.05*x* − 1602.98	0.9999	0.49	1.48
MHEX	0.16∼160.00	*y* = 7100088.19*x* − 21487.66	0.9992	0.05	0.16
BHEX	0.10∼104.20	*y* = 7894602.22*x* − 3054.61	0.9996	0.03	0.10
BOCN	0.12∼117.21	*y* = 5077789.58*x* + 10303.73	0.9998	0.04	0.12
BHEP	0.12∼116.44	*y* = 7970991.13*x* + 29608.09	0.9999	0.04	0.12

**Table 2:** Precision, repeatability, stability, and recovery for the determination of 10 compounds.

Ingredient	Precision *n* = 6 interday (RSD%)	Precision *n* = 6 intraday (RSD%)	Repeatability *n* = 6 (RSD%)	Stability *n* = 6 (RSD%)	Accuracy (*n* = 6)	
					Recovery %	RSD %
BGLU	1.44%	1.32%	1.83%	1.76%	99.58%	1.20%
MGLU	1.58%	1.46%	1.57%	1.16%	99.96%	1.26%
MOCT	1.33%	1.38%	1.50%	1.45%	100.87%	2.23%
BOCT	1.22%	1.42%	1.77%	1.08%	104.39%	1.39%
MOCD	1.27%	1.37%	1.61%	1.23%	101.17%	1.30%
BOCD	1.56%	1.23%	1.97%	0.99%	103.11%	1.43%
MHEX	1.25%	1.12%	1.67%	1.10%	101.99%	1.20%
BHEX	1.53%	1.36%	1.94%	1.02%	99.79%	1.25%
BOCN	1.49%	1.50%	1.80%	1.09%	98.43%	1.22%
BHEP	1.46%	1.18%	1.76%	1.74%	99.41%	1.40%

**Table 3:** Quantitative results for 10 components in maca (unit mg/g).

Ingredient	S1	S2
BGLU	8.4751	9.0742
MGLU	1.8034	2.0923
MOCT	0.5446	0.4337
BOCT	0.5137	0.8825
MOCD	0.2635	0.2525
BOCD	4.5074	5.0337
MHEX	0.0864	0.0652
BHEX	1.0037	0.9863
BOCN	0.2325	0.3287
BHEP	0.0838	0.2136

*Note*. S1: first batch of maca samples; S2: second batch of maca samples.

**Table 4:** Method validation for the determination of five components.

Component	Linear range (*μ*g/mL)	Regression equation	*R* ^2^	LOD (*μ*g/mL)	LOQ (*μ*g/mL)
BISO	0.46–457.23	*y* = 114754.60*x* − 394043.9	0.9990	0.15	0.46
MISO	0.61–606.34	*y* = 120456.58*x* − 62455.57	0.9999	0.20	0.61
MTHI	0.55–551.46	*y* = 101419.60*x* − 15288.8	0.9999	0.18	0.55
CAM	0.50–501.40	*y* = 167256.01*x* − 33240.29	0.9996	0.17	0.50
SIT	0.52–522.30	*y* = 103282.03*x* + 21151.34	0.9992	0.17	0.52

**Table 5:** Precision, repeatability, stability, and recovery of 5 components.

Component	Precision *n* = 6 (RSD%)		Repeatability *n* = 6 (RSD%)	Stability *n* = 6 (RSD%)	Accuracy (*n* = 6)	
	Interday	Intraday			Recovery%	RSD%
BISO	1.09%	1.17%	1.80%	1.19%	99.43%	1.82%
MISO	1.16%	1.24%	1.94%	1.29%	109.99%	1.90%
MTHI	1.12%	1.20%	1.97%	1.12%	103.11%	1.63%
CAM	1.21%	1.14%	1.76%	1.02%	96.79%	2.25%
SIT	1.20%	1.18%	1.57%	1.18%	109.39%	2.39%

**Table 6:** Contents of five components in maca (unit: mg/100 g).

Compound	S1	S2
BISO	2.2645	2.2234
MISO	0.4134	0.3662
MTHI	0.2654	0.1463
CAM	1.0145	0.9842
SIT	4.5154	4.8862

*Note*. S1: first batch of maca samples; S2: second batch of maca samples.

**Table 7:** The contents of 5 components in samples processed via different drying methods.

Group	Content (mg/g)				
	BISO	MISO	MTHI	CAM	SIT
1sg	0.0115	0.0012	0.0045	0.0011	0.0042
140	0.0121	0.0013	0.0047	0.0011	0.0042
160	0.0135	0.0014	0.0053	0.0013	0.0049
180	0.0150	0.0016	0.0059	0.0014	0.0055

*Note*. 1sg: 1 mm thickness, naturally dried; 140 : 1 mm thickness, dried at 40°C; 160 : 1 mm thickness, dried at 60°C; and 180 : 1 mm thickness, dried at 80°C.

**Table 8:** Contents of 10 components in samples processed under different drying methods.

Group	Content (mg/g)									
	BGLU	MGLU	MOCT	BOCT	MOCD	BOCD	MHEX	BHEX	BOCN	BHEP
1sg	1.8042	8.4721	0.5365	0.5063	0.2653	4.5045	0.0765	1.0026	0.2358	0.0804
140	1.8944	8.8957	0.4936	0.4557	0.2520	4.4144	0.0750	0.9825	0.2311	0.0732
160	8.3174	20.1979	0.1610	0.1519	0.0796	1.3513	0.0229	0.3008	0.0707	0.0241
180	9.2415	19.4421	0.0537	0.0506	0.0265	0.4504	0.0070	0.1003	0.0226	0.0080

*Note*. 1sg: 1 mm thickness, naturally dried; 140 : 1 mm thickness, dried at 40°C; 160 : 1 mm thickness, dried at 60°C; and 180 : 1 mm thickness, dried at 80°C.

## Data Availability

The data used to support the findings of this study are available from the corresponding author upon request.
